# Korean Music Therapy Students’ Experience of Group Music Therapy: A Qualitative Case Study

**DOI:** 10.3389/fpsyg.2019.00636

**Published:** 2019-03-29

**Authors:** Hyejin So

**Affiliations:** Department of Creative Arts Psychotherapy, Jeonju University, Jeonju, South Korea

**Keywords:** Korean music therapy students, group music therapy, personal and professional developments, musical experience, cultural context

## Abstract

The purpose of this qualitative case study is to describe the in-depth experiences of Korean students undergoing group music therapy. Seven students participated in eight consecutive weeks of group music therapy. The researcher collected and triangulated three data resources: individual interview transcripts, participant journals, and audiotaped sessions. The data were analyzed using the case study method and peer debriefing was conducted for trustworthiness. The four emergent themes and six categories were as follows: (1) Discovering one’s self (categories: what it means to be “me,” learning about self), (2) Inside group music therapy (categories: various responses to experiencing non-directive group, two different sides of experiencing silence, feeling a sense of safety and acceptance, sharing difficult experience with one’s own family), (3) Musical experience as a way to explore emotions, and (4) What learned through books became real. The results indicated that the student therapists’ experiences were linked to their personal development, unique experience of group music therapy dynamics, musical experience as an emotional container, and clinical development. Finally, it is recommended that cultural adaptations are made by providing both structure and space for Korean students to express themselves.

## Introduction

The notion of personal therapy for therapists in training has been little known in the field of music therapy in Korea despite its benefits for students’ personal and professional growth. The concept of personal therapy for psychotherapists was proposed by Freud in the early 1900s. Freud claimed that analysts would benefit from undergoing analysis themselves (Rake and Paley, 2009). Since the inception of psychoanalytic training, it has thus been considered essential that psychotherapists undergo personal therapy as part of their professional and personal development (Gold and Hilsenroth, 2009). For example, the following types of mental health professions stressed the need for personal therapy: training of psychoanalysts (Caligor, 1985), psychologists (Holzman et al., 1996), family therapists (Forman, 1984; Patterson and Utesch, 1991), and psychiatrists (Habl et al., 2010).

From a learning perspective of personal therapy, trainee therapists can better understand the clients’ position from various angles through the process of therapy (McEwan and Duncan, 1993; Yalom, 2002). In addition, the actual experience of personal therapy enables student therapists to observe their therapists’ clinical skills, approach, and therapeutic application of theoretical knowledge (Rake and Paley, 2009). The students can also model therapists’ “style, attitude and manner.” Interestingly, Gold and Hilsenroth (2009) found that student therapists that had engaged in personal therapy rated their therapeutic relationships more highly and felt more self-assured in helping and collaborating with their clients. Therapists that engaged in personal therapy did not appear to have any conflict in sharing equivalent therapy goals, felt more self-assured, and possessed devoted clients that were convinced of the positive effects of therapy. Indeed, clients with therapists who had received personal therapy remained in therapy for double the amount of time (Gold and Hilsenroth, 2009).

The positive personal developments through trainees’ own therapy course have been discussed in the following studies. Murphy postulated four stages that student counselors face during their own therapeutic process: “reflexivity” as the growing awareness of personal issues triggered during training or clinical work, “growth” as the increasing empathetic understanding of the clients, “authentication” as validating the self as therapist and building positive values of psychotherapy, and lastly “prolongation” as students’ evaluated the necessity for more than 40 h of personal therapy for the sake of cultivating professional skills regardless of whether or not they were experiencing problems. The researcher explained that mandatory personal therapy positively influenced the student counselors through one of these four stages in therapy (Murphy, 2005).

Nonetheless, Edwards (2017) pointed that undergoing a personal therapy simultaneously as a client and a clinician could be a risk as it can cause emotional distress and blurry boundaries. Therefore, she stated that this experience should not be forced, but should be entirely the voluntary choice of students. Additionally, she suggested that training faculty and affiliated association need to openly discuss this issue (Edwards, 2017).

Freud maintained that all psychoanalysts and trainee analysts should undergo personal analysis. Studies also showed that therapists with psychoanalytic and psychodynamic orientations continue to consider personal therapy more important than do those who practice behavioral approaches (Norcross et al., 1988; Bike et al., 2009). Guy et al. (1988) reported that, of all types of therapists, psychodynamically oriented therapists participated in personal therapy for the longest period of the time both before and after becoming professionals. The authors reported that three main motivations to seek personal therapy were: “requirements of the training programs selected by these individuals, greater personal need, or general introspective tendencies” (p. 475). On the other hand, therapists who practice in the field of Cognitive Behavioral Therapy (CBT) generally do not consider it as important to receive personal therapy as a client. Only recently has the idea of incorporating personal therapy gained momentum in Europe, as CBT mainly seeks to promote changes by encouraging clients to learn and apply practical skills as a treatment tool (Geller et al., 2005). Clients are also encouraged to enlist their family and friends and to develop strength and coping mechanisms (Norcross et al., 2009). Linley and Joseph (2007) showed that therapists who used cognitive behavioral approaches reported experiencing fewer personal changes but more feelings of exhaustion.

Music therapy is a discipline that uses music for therapeutic purposes, supporting diverse client populations in improving their physical, psychological, and cognitive function through a therapeutic relationship (Wigram et al., 2002). Different types of music therapy have different theoretical foundations, and training programs include behavioral approaches, psychotherapeutic and psychoanalytic approaches, client-centered therapy, and transpersonal therapy. To become a competent psychotherapist requires “maturity derived from a depth and range of personal life experiences,” as well as clinical skills (Lister-Ford, 2007, p. 112). A student music therapist or professional music therapist needs to continually evaluate him or herself objectively to keep growing and developing. Pursuing personal therapy can be one of the greatest ways for music therapists to continue to grow and develop (Bruscia, 1998; Wheeler et al., 2005). Especially for psychodynamically oriented music therapists, undergoing personal therapy is essential to understanding one’s unconscious and its influence on clients (Hadley, 2003).

For music therapy students, the direct experience of group music therapy can be “also excellent for opening up creative potential and exploring the possibilities of music therapy” (Tims, 1989, p. 92). Jackson and Gardstrom (2012) indicated that after participating in a group music therapy, students reported increased self-insight, experienced emotional support, enhanced relationships with others, and cultivated musical creativity and clinical skills. Likewise, Fox and McKinney (2015) reported that receiving Guided Imagery in Music sessions enabled interning music therapists to increase their self-confidence and emotional understanding. In terms of professional changes, the participants came to understand the importance of the therapeutic process and space and developed a musical relationship.

A literature review indicated that only two institutions—New York University and Alborg University in Denmark—require that students undergo personal therapy as part of their music therapy training. Hesser (1985), of New York University, developed an advanced music therapy training program that includes in-depth music therapy. At New York University, master’s degree-seeking students are required to participate in weekly group music therapy as a client for 2 years. Hesser (1985, 2001) strongly believes that this mandatory personal therapy enhances students’ personal and professional growth and communicates the strength and effectiveness of music therapy work. For students to feel safe, program administrators make an effort to create “a non-competitive” (Hesser, 1985, p. 68) atmosphere in which grades are not as important as students’ participation. In this type of program, Hesser (2001, p. 55) feels that “the group process has become a central and important part of the training program. The music therapy program at Alborg University in Denmark is based on humanistic and psychodynamic theories. While this program also requires students to receive either verbal psychotherapy or music therapy, this is done outside of the training program by non-affiliated therapists, due to ethical concerns around having one’s instructor also be a personal therapist, or having to relate to classmates as group therapy co-participants. The goal of experiencing music therapy as a client is “the acquisition of therapeutically flexible musical skills” (Wigram et al., 2002, p. 271).

Despite the benefits that these two programs see in requiring music therapy, concerns have been raised about requiring trainees to undergo personal therapy. The American Music Therapy Association (AMTA) does not mandate that trainees receive personal therapy as a client, leaving this option up to the directors of individual programs (Jackson and Gardstrom, 2012). Gardstrom and Jackson (2011) reported that most of 41 music therapy directors in their study avoided mandating personal therapy for students, except when students manifested serious personal crises or mental illnesses that interrupted or compromised their training experience. Although multiple studies have emphasized the importance of personal therapy as a client, none examine students’ actual experience of personal therapy as a client. Jackson and Gardstrom (2012) further stated that not everyone is willing to pursue self-exploration, and call for more research considering the degree level of specific programs, students’ developmental levels, and program directors’ previous personal therapy experiences to better understand the influence of personal therapy on training music therapists.

Some post-graduate music therapy training programs also require personal therapy. For example, Analytical Music Therapy (AMT) uses “analytically informed symbolic use of improvised music” (Priestley, 1994, p. 3) in the therapeutic relationship. This approach aims to explore clients’ inner selves and help them to gain self-insight, with the client’s emotions and unconscious explored through sound expression. Therapist countertransference plays a role in connecting with each client’s repressed feelings (Priestley, 1994). Trainees in the field of Analytical Music Therapy (AMT) must undergo both individual therapy with an AMT therapist and intertherapy with another trainee, as a pair. During individual therapy, trainees both learn clinical techniques from the therapist and discover their own inner life with the aid of shared music and words (p. 297). Priestley (1994, p. 287) maintained that this experience is only way for trainees to understand “[their] own patients and the right sensitivity and care in the therapeutic use of this powerful art form.” During inter-therapy trainees function as therapists for their fellow trainees, and develop their own personal style of therapy, learning how to connect and work with clients.

To give another example, Guided Imagery and Music (GIM) is a receptive form of music therapy in which the client listens to classical music and connects with the imagery that they associate with it (Wigram et al., 2002). In GIM sessions, each client’s experiences are viewed through a psychodynamic and transpersonal lens (Summer, 1998). GIM training entails receiving a certain number of GIM sessions for each level of training. This experience is intended to promote trainees’ professional and personal development, helping trainees to learn the “skills and knowledge needed to engage clients in psychotherapeutic music-imagery process” (Abbott, 2013, para. 5).

Finally, Nordoff-Robbins Music Therapy was influenced by humanistic and psychodynamic approaches (Turry, 1998; Wigram et al., 2002). The basic belief of this approach is that everyone has the innate musicality needed to respond to music (Nordoff and Robbins, 2007). In this modality, musical improvisation is a significant channel through which to interact with clients, emphasizing “immediacy, involvement, [and] unpredictability” (p. 27) and promoting the “qualities of awareness, listening, and creativity” (Ansdell, 1995, p. 27). While Nordoff-Robbins training is designed to enhance each trainee’s development through “a combination of supervision, classes, and clinical work,” trainees are expected to undergo personal therapy as part of their training in the future (Sorel, 2013, Para. 1).

Although a small number of studies have addressed personal therapy for student music therapists in Korea, these were only a part of the class experience (Moon, 2014), professional development (Bae, 2011), or served as brief introduction to the benefits as part of the outcome of such therapy (Lim and So, 2017). Therefore, the present study aims to conduct an in-depth investigation of the experience and participation of Korean music therapy students’ undergoing a group music therapy. The proposed research questions are as follows: (a) what did Korean music therapy students experience inside the group music therapy? (b) how did the group music therapy help Korean music therapists’ personal and professional development? (c) how did cultural expectations influence Korean music therapy students in their group music therapy process?

## Materials and Methods

### Research Design

This qualitative study used the case study approach to explore and understand Korean music therapy students’ experiences of 8 weeks of group music therapy in-depth. To examine their inner process, dynamics, and clinical developments, the case study method was selected because it allows for the in-depth exploration of the participants’ experiences (Crowe et al., 2011) as cases “independent of any particular investigation” and “bounded” context (Schwandt and Gates, 2018, p. 342). Moreover, the case study method is effective at examining questions concerned with “how,” and “why” (Yin, 2014) and provides “a variety of lenses which allows for multiple facets of the phenomenon to be revealed and understood” (Baxter and Jack, 2008, p. 544).

### Recruitment

Purposive sampling was used to recruit research participants. Upon approval of the Institutional Review Board of Lesley University, the researcher visited a music therapy master’s program in South Korea with which the author was not affiliated. While the author was preparing this study, the director of the music therapy department at this university contacted the author to request that she lead a music therapy group for her students. The author proposed this study plan and the director accepted it. The author visited the director’s class and gave a 20-min presentation about the study to recruit potential participants. After this, the author provided a verbal explanation and a written informed consent form that included a description of the purpose of the study, the possible benefits of participation in the study, participants’ right to withdraw from the study at any time, and the protection of confidentiality. In addition, possible difficulties during group music therapy were outlined in the informed consent form. These possibilities included emotional reactions, assuring participants that the author would provide resources and referrals if needed. Seven female and two male students provided written consent to participate in eight consecutive weeks of group music therapy, and copies of the signed consent forms were given to all participants at the first session. At the time of the study, seven of the participants were enrolled in the second semester of their graduate program and the remainder were in their first semester. Participants ranged from 24 to 32 years old. The pre-survey indicated that five participants never received any type of personal therapy before. Three participants had undergone informal counseling with pastors or short-term counseling sessions (see Table 1). One participant had been receiving individual Guided Imagery and Music sessions for eight months. In the middle phase of the group music therapy, both male participants withdrew owing to family emergencies.

**Table 1:** Demographics of the participants.

	Gender	Age	Semester enrolled	Previous therapy experience	Type of previous therapy received	Duration of therapy received
A	F	26	Second	No	N/A	N/A
B	F	24	second	No	N/A	N/A
C	F	26	Second	Yes	Individual guided imagery and music	8 months
D	F	24	Second	Yes	Individual Christian counseling	Once every 2 months for 2 years
E	F	24	First	Yes	Typical counseling	6 session
F	F	25	Second	Yes	Pastoral counseling	Not on a regular basis
G	F	24	Second	No	N/A	N/A

### Data Collection and Data Analysis

As Creswell and Poth (2018) suggested the use of “multiple sources of information” (p. 96) for a case study, the researcher triangulated three different types of data resources: audiotaped sessions, individual interview transcriptions, and participants’ weekly journals. The researcher audiotaped each session and documented significant musical playing and verbal discussion in chronological order. Thereafter, participants were asked to record weekly journals on their thoughts and feelings of group music therapy. Lastly, the researcher conducted semi-structured interviews with each participant individually within 6 days of the termination of group music therapy (see Table 2 for interview questions). The duration of the shortest interview was 32 min 59 s and the longest interview was 56 min 28 s. The researcher transcribed all the interviews verbatim.

**Table 2:** Interview questions.

General questions	(1) How was the group for you?
	(2) What were your expectations for this group?
	(3) What was your most difficult experience?
Personal development	(1) What was your most meaningful personal experience in this group?
Professional development	(1) What was your most meaningful experience as a music therapy student?
Musical experience	(1) How did the music influence you?
	(2) How did the music help you to reflect?

The process of qualitative data analysis involves “examining, categorizing, tabulating, testing, or otherwise recombining evidence, to produce empirically based findings” (Yin, 2014, p. 131). However, there is no proscribed method of data analysis for case studies, a method for which data analysis is “one of the least developed aspects” (p. 133). Yin (2014, p. 132) therefore suggested that researchers focus on looking for “patterns, insights, or concepts” during data analysis. In light of these suggestions, the researcher modified and used a simplified version of Moustakas’s (1994) method by Creswell (2013), which provides “the most practical, useful approach.” In the present study data analysis was conducted as follows: (a) highlighting significant statements in all three data resources (b) reading significant statements numerous times and noting possible themes (c) listing significant statements and formulating meaning for each significant statement (d) categorizing significant statements and grouping them by theme (e) identifying and prioritizing more significant statements for each theme and eliminating statements that appear as weak evidence for each theme (f) produce in-depth descriptions of participants’ experience grounded in the themes and categories.

### Trustworthiness

To establish the trustworthiness of this qualitative case study, the researcher triangulated verbatim interview data, participants’ weekly journals, and written summaries of each audiotaped session. In addition, the researcher employed peer-debriefing throughout the process of coding and finding themes and categories. The peer in consideration was also enrolled in a doctoral study in a related field with experience in conducting several qualitative research studies. Additionally, the researcher periodically consulted with a doctoral advisor throughout the research projects. Both of these processes, with a peer and an advisor, enabled the researcher to be objective in this coding process. For example, to extract the theme related to participants’ various reactions toward a non-directive group the researcher discussed this theme with her advisor and was able to view and interpreted this category in a Korean cultural context, adding a crucial element of richness to this study.

### Researcher Reflexivity

The author was trained in a humanistic and psychodynamic tradition as a music therapist in the United States. She is a certified Nordoff-Robbins Music Therapist and completed a 1-year psychodynamic clinical practice program at a psychoanalysis institute in the United States. At the time of this study she had 7 years of clinical experience. The music therapy program that the researcher attended in the United States included group music therapy as a part of the curriculum. As an international student from Korea, participating in the group music therapy served as vital support in the 2nd year. After returning to Korea, this experience has been a continuous positive influence on her teaching, practicing, and supervising. Although, the 1st year of the music therapy group was difficult because this therapy was new experience for her. The researcher desired to introduce the experience of group music therapy to students in Korea because it would be beneficial for them. However, the researcher sought to examine how the cultural gaps could be identified and adapted appropriately for Korean students as she experienced in the United States. In the process of leading the group, the researcher kept her own journal to write about the thoughts, feelings, memories, and dreams that occurred during the study; she also received personal supervision from a music therapist with over 20 years of experience in both teaching and clinical practice. These two practices played an important role in revealing and managing the author’s countertransferential reactions toward participants, and in avoiding overidentifying with them.

### Context of Case

The researcher served as the group leader and conducted eight consecutive weeks of group music therapy. Because the participants in this study did not have pressing clinical needs or goals, the author established therapeutic goals related to gaining insight about the self and “awareness of issues, thoughts, feelings, attitudes, and conflicts” (Wigram et al., 2002, p. 31). Group music therapy consisted of musical improvisation and verbal discussion. Improvisation in a music therapy context means that clients create music spontaneously through “playing an instrument or singing, extemporaneously creating sound forms, melodies, rhythms, or entire pieces” (Bruscia, 1998, p. 5).

The group met every Wednesday morning for approximately 60 min in their classroom. There was a black grand piano on the left-hand side of the classroom and various musical instruments were stored directly outside of the classroom. The participants selected and brought in any instruments they desired to play before the group started. Examples of the instruments made available were a snare drum, small percussion instruments, cymbals, guitar, ocean drum and a metallophone; participants could also vocalize. The group sat on the floor in a circle and the instruments were placed in the middle of circle or in front of each member.

The leader facilitated the group with an emphasis on the “here-and-now,” but welcomed any topics related to past experiences, memories, or difficult relationships that participants wished to explore or discuss. Group music therapy was led in a non-directive way, with the leader providing minimal structure. The group usually started with simple stretches for relaxing and a musical check-in. Playing music usually occurred both at the beginning and the end of each group, with a verbal discussion during the middle of the group. During musical check in, a projective technique was used, with each group member expressing their feelings, thoughts, and concerns through improvising with their instrument. During musical play and verbal sharing, common themes were identified and selected to explore during that session. For the end of each session, the leader led a calming and containing musical exercise if needed. To facilitate verbal processing and to help participants to integrate their musical experiences, the leader would ask questions such as “What feeling did you just play?” If somebody made a comment or brought up an issue, the leader redirected it to the whole group and asked, “How do other people think or feel about the comment that was just made?” This framing encouraged group members to become more interactive and to avoid focusing on only interacting with the leader.

#### Session 1

The leader suggested free improvisation to start and explore the session. After some of members asked if they could start any time the group started an improvisation. In the first improvisation, the group played only rhythmic instruments, finding basic beats very quickly but playing without dynamic changes. After a while, music was played in a faster tempo and more loudly. As the group subdivided the rhythms more, the music sounded more intense. After the first improvisation, the group remained in silence for a while but started to improvise soon. During the second improvisation, some of the group members played melodic rhythms on instruments such as piano, metallophone, and guitar. With melody being played, the music sounded more structured. One participant went to the piano and began to play in a blues scale; another played on a metallophone. One participant began to play the guitar in a major key, but to a different beat than the rest of the group. One of the female members sang the notes of the chords on the guitar. Everyone laughed, and the music stopped. After the improvisation was ended, the group shared their experience with free improvisation. Because free improvisation was very new experience for most of the members, some were curious about what would happen next; others reported that it was hard for some of them to start music without direction. This experience made them feel more conscious of other people and made them want to depend on others as well.

#### Session 2

The group had a very long verbal discussion. The group members had different ideas about the group. Some of them felt excited to come to the group but the others felt less comfortable in the group and overly conscious of others. After one member asked a question about how others chose their musical instrument the group shared their connections between their thoughts and instruments. Another important discussion occurred, as the group began to explore verbally what they wanted to achieve in the group. This discussion was an opportunity to get to know each other better. When the leader reminded participants that the group was about to end after a long verbal discussion, one of the members suggested that they play music. The group began to play music slowly. When one member began to play a four-beat harmonic progression repeatedly on the guitar, the rest of the group played along. The member who was playing the guitar lead the group music. The group sustained playing at a moderate tempo for a while. When one of the group members initiated playing the tremolo, the rest of the members also played tremolo and slowly ended the music together.

#### Session 3

The group talked about different feelings, thoughts, and related memories relating to silence and to the desire to have the “correct answers” in the group. After the discussion, one of the members asked the leader if she could suggest that they music all together. The participant added that she wanted to play her instruments. While the music was not harmonious at the beginning it became cohesive, with a fast tempo and sustained playing to the basic beat. The music sounded as if the group members were letting out their energy cathartically. The leader also played the drum very strongly and loudly to support this group music. When one participant suddenly played a short phrase on the metallophone the group gradually stopped playing.

#### Session 4

The leader started the group with a musical check-in in which everyone took a turn playing music solo and had an opportunity to share their feelings and thoughts. Through this activity, the group members shared their fear of interrupting others in the group, wanting to play music very loudly, worries about family, and difficulties in relationships. Members shared feelings such as sadness and anger more deeply in this session than in previous sessions. When one of the members shared difficulties in expressing her anger, the leader suggested that the group support her in letting out her anger. At this time, as the leader initiated beating the drum very hard the group joined in, beating the drums and percussion instruments very hard and loudly. In the middle of the drum playing, the leader played a dissonant chord to support the group at the piano. When the group playing had more space and was less intense, the leader gradually moved into a melody in minor on the metallophone, so that a member who shared her sadness could also be comforted and feel accepted in the music.

#### Session 5

The members revealed personal stories and became emotional for a long period of time. The leader shared with the group that everyone had some struggles in life and suggested playing music “only for myself.” The group accepted the leader’s suggestion. The group started playing music in a moderate tempo. One of the members improvised melodies on the metallophone in a minor scale. Another member began to play the piano in a lively major key with various melodic patterns. Music was played in an upbeat and even tempo. After playing, the group shared how their needs were projected and supported in group music.

#### Session 6

The members verbally expressed trust toward each other and talked about their family relationships. After a long verbal discussion, the leader asked what they wanted in music before the group ended. One member answered, “harmonious music.” Another member said, “music will become harmonious once the group started to play.” In this improvisation, a few members played ocean drum and a rainstick (both of which have no pulse). Someone played the basic beat on the drum, which was grounding. Other filled the spaces with eighth and quarter notes. The specific rhythm patterns on the drum and short melodic phrases on the bells were played repeatedly in a call and response form for a few minutes. The melody in a pentatonic scale on the bells had more notes and leaps between notes.

#### Session 7

When the group started stretching the leader encouraged them to make natural sounds with vowel such as the “ah” sound. The leader reminded the group that they would be terminating in the next session. The members talked about feeling sad about terminating the group and shared that they were surprised about how much they were able to disclose in the group. They also continued to talk about difficulties in their families. As some of them shared how they were not free and held back from their needs, the leader suggested that they play music as they wanted without being conscious of others. The group then directly requested to play harmonious music together. The group music was very cohesive. The rhythms were subdivided at times but still in the same tempo and on the same beat. Vocalizing emerged for the first time in the group. When the leader initiated vocalizing one of the members repeated joined in, but stopped vocalizing with the leader several times. After the group improvised music loudly and intensely for a while, each instrument faded out and only bell and ocean drum were being played very quietly.

#### Session 8

The members wanted to end the group “like a party,” and wanted to do a musical celebration to complement each one’s accomplishments and changes throughout the group process. Every time each member received complements on his or her accomplishments, the rest of group members improvised with their instruments briefly, shouting with delight. After everyone was done, the group also thanked the leader. The leader responded to the group by improvising a poem with images of a blossoming flower. When the leader asked what kind of music they wanted to play, the group wanted to play music as it flowed. The group started improvising without hesitation and the music became very rhythmical. Later they played like a group drumming powerfully. The group ended after a short conversation about how they felt about the music that they had improvised.

## Results

Four themes and six categories emerged from the data analysis: (1) Discovering who I am (categories: what it means to be me, learning about self), (2) Inside group music therapy (categories: it was flowing like water, silence, I can do whatever I want safely here, and the truth of my family), (3) The role of music, and (4) what we learned through books became real (see Table 3). In the description of results, all the participants’ names were disguised to protect their privacy.

**Table 3:** Theme, categories, and examples of quotations.

Theme (4)	Categories (6)	Examples of quotations
Discovering one’s self	What it means to be “me.”	“I was able to show my introverted self without difficulty.”
	Learning about self.	“I was surprised to hear compliments that I had not expected from the group.”
Inside Group Music Therapy	Various responses to experiencing non-directive group.	“You did not give us any specific directions. Yet I was surprised to see [the group] was flowing like water without any specific direction.”
	Two different sides of experiencing silence.	“Silence was uncomfortable.” “I liked that the moments of silence allowed me to reflect on myself.”
	Feeling a sense of safety and acceptance.	“I can do whatever I want safely here.”
	Sharing difficult experience with one’s own family.	“I couldn’t express my needs because they might worry.”
Musical experience as a way to explore emotions		“As it was hard for me to talk, I reflected [my feelings] through the musical instruments.”
What learned through books became real		“I feel like what we learned through books became real and did not remain as just theory.”

### Theme 1: Discovering One’s Self

The first theme consists of two categories (1) what it means to be me; (2) learning about self. Both categories describe how students began to individualize and develop personal awareness.

#### What It Means to Be “Me”

Participants reported becoming more focused on their own feelings and thoughts and away from other people’s needs. The following statement illustrates an example of participants B’s awareness of intrapersonal distance at the beginning: “I did not even know what I was thinking; … I did not know what I was feeling. …I had nothing to express.” However, she said that she became centered in herself and “more honest in expressing feelings” and “Now I began to express a little bit more about what I am unhappy about.” However, she labeled this change as “being selfish.”

Participants G and A stated that they discovered hidden parts of themselves. Participant G had viewed herself as an extroverted person but found introverted parts of herself that she did not want to reveal to other people. Facing hidden sides was “awkward” and “a little confusing” because she wondered which side of her was more genuine. In the session, she openly discussed that “Once I opened up myself, I knew group members would accept it, so I was able to show my introverted self without difficulty.” Likewise, participant A, identified a gap between the parts of herself that had to adjust from needing a positive evaluation from other people. In a couple of reflective journals, she processed this issue closely: “I was always busy being conscious of other people. …This group provided me an opportunity to concentrate on myself and enabled me to realize exactly what it means to be me.” In her following journal, she wrote: “I had always avoided this because I did not want to admit that there were big differences between my real self and my ideal self. … since I began this group music therapy I have realized how important it is to face it.”

#### Learning About Self

The participants were able to gain new insights about themselves through verbal discussion or feedback from group members. Participant F detailed the impact of other group members’ comments on her in her personal journal – “I was surprised to hear compliments that I had not expected from the group. In general, people say to me that I am committed and nice. That is all. However, participant G said she saw different parts of me in the session. Participant A told me I am determined… I was surprised that they noticed it. I was grateful that group members recognized and discovered this side of me.” Both participant D and G reported learning about their repeated role serving as caregivers throughout the group. Participant D felt responsible easily and took things on herself if something happened in general. “Even though it is not me, there is always somebody who can do it. …. So, I was able to let go of the pressure about taking responsibility.” Similarly, participant G believed that people probably believed she would take the initiative and admitted feeling that it was “a burden” for her to take responsibility in any type of group. She shared her awareness of feeling responsible frankly – “I think I want to prove my sense of self by doing something. I want other people to remember me. I want other people to feel how empty it is if I am absent and I want other people to miss me when I am not there.” Participant G also expressed her anxiety about this new insight in her personal journal: “Afterwards, I came to realize there are some problems in my relationship with my mom, with whom I had thought I got along fine for the last 25 years. I felt lost and scared. It is the first time that I felt scared of therapy. I am scared that my life, personality, and thoughts may change completely after I find and resolve the root cause of this [issue].” It appeared that she developed anxiety about approaching new areas to explore more deeply as this was her first experience of therapy.

### Theme 2: Inside Group Music Therapy

This second theme directly discusses Korean music therapy students’ internal experiences and discussion topics during group music therapy.

#### Various Responses to Experiencing a Non-directive Group

At the beginning phase of the group, participants struggled because participating in a non-directive group was unfamiliar to them. They commonly reported a need of structure and clear direction of what to do in the group. Participant A described the session format as follows: “it is not like my time was set in 10 min [to do an activity] or improvisation. How we participated changed a little bit every time. Everything was new.” In the first weekly journal, she wrote about expectations of the group leader that “I wanted to depend on the group leader a lot. When we were doing nothing, I wanted her to make a decision for us.” Participant B stated in the interview that “It would have been easier and more comfortable if [you] had given us directions and told us what the first, second, and third activities were.” In the first journal, she illustrated “I felt that I needed to find the structure on my own. I wish somebody had told us when to begin and end the music.” Participant C wrote upfront about her impression of the leader in the first journal. “I thought that the group leader was not engaged [with us] that much and did not function.” She shared in the group that “I kept being engrossed in wondering if what I did was right or wrong…. I thought I needed confirmation and concrete reassurance.” In the interview she said that “I kept looking at you to see if you would give us some direction. Similarly, participant F stated that. “[You did not say] ‘it is the next person’s turn’.”

Positive experiences began to emerge gradually from the middle of the overall group process. Participant B reported in the interview – “You offered questions that we could reflect upon ourselves. I really liked it very much when you asked how we were feeling and how we were doing on that day.” While participant C recorded that – “The leader makes minimal interventions. However, when we think, she offers questions at the right moment that makes important points that lead us to contemplate and to discover our own answers.” Participant D reported that she learned about the role of the group leader in the interview: “I learned a lot – that despite the fact that the leader was not involved in every single moment, the group could still have [their own] dynamics. Furthermore, people were capable of disclosing personal information.” She added more in the journal. “I was able to observe that the leader helped us to express emotions and think more deeply. I think the leader’s role is to help people feel less pressured, talk naturally, and feel safe. The group leader’s role was to listen actively to what the members were talking about, and to make decisions about how to respond to the members, how to lead the group, and how to accept the closing of the members’ discussion.”

Participant E shared a similar observation on the group leader’s role in the interview – “You did not give us any specific directions. Yet I was surprised to see [the group] was flowing like water without any specific direction. Also, it was surprising to see that a structure developed independently with only a small number of directions.” Participant E’s following statement represents an account of the beneficial nature of the group – “I think I was able to be more honest in expressing myself. I think I was able to be free. If there was a fixed direction from the beginning of the group, I would have tried to fit my thoughts in that direction and it would have resulted in dishonest results [or responses]. It was confusing at the beginning; however, it was meaningful for me that I was able to open myself up.”

#### Two Different Sides of Experiencing Silence

Silence reflected various aspects of the participants’ experience. Being silent was an overwhelming experience for many participants, especially at the beginning of the group. Participants described how silence provoked anxiety and made them conscious of the group leader and other members. In the second, third, and seventh sessions, silence was an important topic of group discussion. For example, Participant E stated in the interview, “to be honest, silence was uncomfortable.” She also said that during times of silence she hesitated to speak because she was shy. Both Participants B and C reported that they became conscious of others’ eyes in the group. Participant B was uncertain as to whether she should break the silence as, although she desired to talk, she worried “somebody might want to talk as well” and she wanted to avoid being intrusive. Moreover, she was worried that the group leader was wasting the time that she had allotted to conducting research and that she would gain nothing from the group because there was too much silence. Participant C reported that she had to break the silence because she generally experienced silence as awkward and intolerable. She approaches people first when meeting for the first time and this tendency was reflected in the group process as well. Participant C reported that she was “afraid of breaking silence” as she did not want to affect other people. Participant D shared candidly that she “hated” silence and, during times of silence, she began to observe what other people were doing. It was surprising for her to realize how difficult silence was for her and she noticed that she daydreamed to escape the moment instead of focusing on herself. She became aware that she would subsequently feel guilty for not using this time more effectively. Similarly, Participant G wrote in her journal that “I tried to break the silence. Others seemed to feel uncomfortable in the silence itself but the atmosphere that others were feeling uncomfortable made me more uncomfortable. I often feel responsible to resolve and redirect this kind of moment.” Participant F reported she felt timid when silence occurred after talking. This silence led her to believe she might have said something wrong despite being aware that people were processing or waiting for their turn to talk. Participant F further shared both in the discussion and her journal that this silence triggered difficult memories from her childhood. When her mother scolded her, her mother would gaze at her in silence, which frightened this participant. She wrote that “I was surprised that it felt like I was being scolded even though it was just a pause.”

Positive experiences of the silence were shared as well. As the group leader allowed this silence in the group, it served as an opportunity for Participant D to reflect more deeply about herself: “If the group leader asked other kinds of questions or redirected the group in silence, I wouldn’t have time to talk about myself more authentically.” She also added in the interview that the leader’s role is important, as she or he decides whether the silence should be extended for clients to contemplate and process, or whether the silence should be ended. Participant G found the silence necessary. She took this silence as an opportunity for her to gain insight, wanting to discover why she felt uncomfortable and pressured in the silence. Thus, she appreciated how silence enabled her to face herself and explore: “Silence is necessary. It is not a time that we do nothing. We need to learn why silence is difficult. I liked that the moments of silence allowed me to reflect on myself.”

#### Feeling a Sense of Safety and Acceptance

Six of the seven participants mentioned that they felt safe and accepted in the group music therapy. In addition, self-disclosure among group members was one of the significant elements that developed trust within the group to talk about personal issues. Participant A stated she felt comfortable and lighter after talking to the group, described the experience as “sharing secrets with friends” and expressed that other members that were honest in the group motivated her to reveal herself. In the final session, she talked about one of her accomplishments in the group: “I would like to compliment myself that I found courage. Usually being brave or taking [risks] involved regret. However, I did not have any regrets and fear during the therapy. I learned in the class that a safe environment is essential for a client.”

Participant C also reported that other members’ self-disclosure encouraged her to talk about her story. After the group members (herself included) talked about family, she felt closer to and came to understand the members better. In her personal journal, participant F wrote “When participants A and G talked about themselves, I thought they were brave people. Moreover, when I saw other people sharing their feelings, I thought I might be able to do it, too.”

On one occasion after participant G worked on her anger and suggested that the whole group play the drums together loudly in the session, she felt it was like a turning point in trusting the group more. She wrote about this experience in the journal: “I can do whatever I want safely here. Nobody looked weird when I was beating the drums. Also, because everyone played the drums so loud, my loud sound did not stand out. So, I was able to play loud comfortably. This loud sound provided me a safe place even when I cried. Even though it was loud, I felt like that loudness was surrounding me and blocked me from other thoughts.”

Participant D shared how she felt about the members in her journal: “I liked that I spent time with the members that I trust and feel comfortable with. I was also able to become courageous by witnessing other members’ courage and acceptance.”

#### Sharing Difficult Experiences With One’s Own Family

As the group music therapy evolved and established trust over time, participants’ discussions deepened. Members talked about their feelings concerning their parents, their’ specific role in the family, and the dysfunctional sides of their family. In talking about their family, the participants grieved over family that they could not have or expressed anger concerning their family. Participant F shared that “I’ve never seen my parents happy. To me my mom is always a victim and my dad is a perpetrator. Even though he is nice to me, I have always disliked him.” She wrote in her journal that “I unconsciously thought that only my family was in a sad situation but when others talked about their stories [about family], I felt connected and empathic when the other group members expressed different types of difficulties within their family.”

Both participants B and D opened up about their physically ill mothers. In the interview, participant B described the period that she cried talking about her mother who was physically weak due to a low functioning immune system as a meaningful moment. Participant D was surprised to recollect something about her family that she had forgotten during the group discussion. She wrote about her role of being her mother’s caregiver both physically and emotionally. That she used to envy other children who had healthy parents and sometimes felt irritated with the family situation particularly during puberty: “It was not easy to say that I had a hard time, or I was sick because my mom looked more distressed and not well. It became natural that I pulled through things by myself and my parents depended on me.”

Participant C shared a similar experience about expressing her needs to her family: “I couldn’t express my needs because they might worry.” She often felt guilty because she believed that her family had made sacrifices due to her physical illness. However, she “harbored ambivalent feelings” that her mother was overprotective, and her father was “too authoritative.”

In the group, participant A realized that high school clients triggered countertransference in her because she used to feel unable to meet her parents’ expectations. She reported still finding it difficult to accept that she could not please her parents. Since her unhappy family was such a huge issue for her, she had believed that other people’s behaviors were all related to their family backgrounds. She was desperate to receive positive evaluations from other people and had avoided talking about any pain in her familial relationships: “I talked about my relationship with my father and family [in the group]. I had not wanted to talk about this because people might have judged me and pretended to understand me. However, I realized that this viewpoint belonged to me. That is why I was not being honest [about my family] and wanted to present only a happy side.”

Participant A was finally able to let her tears come out. In her personal journal, participant A continued to write about this issue: “Everyone cannot be perfect, right? I used to be very upset and even felt ashamed about my family relationship. I did not cause this.” Participant G courageously talked about her mixed feelings about her family as well. Due to her parents’ divorce and the absence of a father in the family, it was difficult for her to hear other members talking having a good relationship with their fathers: “there is no father in my family. I do not know what the bonds and love is like between father and daughter. I thought I had no anger toward him. But I realized that I am still not fine.” She also reported that it made her feel envy, anger, and turmoil and it was the first time that she had ever spoken about her current family situation.

Even though it was painful for the participants to share their family stories, they had a positive reconstructive group experience through support and empathy from each other. The following statement from participant G’s journal revealed their group cohesion: “I learned the power of group. The question of ‘why did this happen only to me?’ or having feelings such as sadness, and anger made me believe that I was abnormal. …I learned that there is somebody who shared similar experiences and feelings my own. So, it was easier for me to disclose my story. This is exactly what a safe place means to me.”

### Theme 3: Musical Experience as a Way to Explore Emotions

In the group, music functioned to contain, express, discover, or hide the participants’ emotions. Participant E reported being able to identify her feelings right after playing. This became a bridge to exploring her feelings and allowing her to verbalize them. Participant D talked about the benefit of expressing her feelings non-verbally in music. She claimed that music became the most crucial medium to identifying feelings that cannot be dealt with by merely talking. She was afraid that verbalizing them might be wrong. Participant G also maintained that music enabled her to play feelings that were hard to express verbally. Participant C mentioned that music reflected her feelings accurately such that she was able to accept them. In a personal journal, she documented that music provided her with the comfort and space to organize and redefine her feelings. When sharing her sadness, she found that music enabled her to release her feelings honestly, both for her as well as the group.

Participant C stated that music was very helpful at evoking feelings in her. She expressed emotions most of the time while playing music. While listening to her music, she found that emotions that she was previously unaware of rose to the surface. Participant E who appeared to be reserved and quiet in the group mentioned. “As I said before, I could not speak well in the group. As it was hard for me to talk, I reflected [my feelings] through the musical instruments. So, I played the instruments hard to get out any stress that I had from not being able to talk. Or if I wanted to hide [from the group], I brought instruments such as an egg shaker because it had a soft sound. I think music allowed me to express my feelings well.”

There was group discussion on distinctive parts and role that music had compared to typical counseling when participant F raised a question in the seventh session that pointed out that group music therapy did not seem so different from a prior counseling group that she participated in during college. Participant G immediately responded to this doubt by pointing out that she was able to display her anger through loud music, and that she would have had to scream to express herself if there was no music. Participant F wrote about this moment in her journal – “When I asked the group if just talking could have same effect in the group music therapy without music, the group members stressed the power of using music. I was able to agree with them.”

Participant A talked about music being an emotional container: “There was one time that I was about to burst into tears and wanted to calm myself down. It happened when we were about to close the group with music and afterwards, I had verbalized my feelings frankly in the group. If I had not had that [music] time, I just would have wailed.”

Participant G talked about her anger toward things in her life that she could not control. She described her rage as if it would destroy everything. Upon the leader’s suggestion, she invited other people to join in by playing music loudly so that her music would not be particularly distinguishable within the group. She recalled this moment in the interview: “When we played the drum together, expressing rage and anger, I am not sure if this is right, but I felt as if the expression of anger had turned into a pleasant moment. …. Expressing anger became not really anger [in the middle of playing]. I came to enjoy playing all together.” Participant B described this moment in her personal log: “when I played loudly with group to help participant G, I felt like we became one, with one heart, and I liked the fact that we could comfort through music.”

However, resistance to revealing feelings also manifested in the group. Participant A stated that expressing her emotions through music was “too abstract and symbolic” for her. She also indicated that music functioned as a defense that enabled her to hide her true feelings. For example, even though she felt depressed, she intentionally played bright and happy music to hide it. She expressed that she found it “ironic” that she played happy music when she was feeling sad. Participant F mentioned her difficulty in expressing her feelings as well. She associated musical expression with stereotypical playing rather than as a vehicle for expressing her own feelings. “I tried so hard, but my playing came out by habit. If I was sad, I just played slow music and if I was anxious, I played fast music. I played music based on the way I was educated. So, I just played the feelings I had learned.”

### Theme 4: What Was Learned Through Books Became Real

This theme discusses how the practical experience as a client in the group promoted participants’ understanding of their academic and clinical work. Participant G’s statement characterizes this theme: “I feel like what we learned through books became real and did not remain as just theory.” She wrote in her final journal about her mother transference toward the group leader: “I had wondered how a client could become attached to a therapist. Now I finally can see how I became attached to the group leader, who does not look like, has different personality from, and is even not in the same age range as my mom.” Participant D stated “I learned about dynamics, transference, and countertransference in an improvisation class. Now I can understand them much better because I experienced them.” She further stated because she experienced theories rather than acquiring such learning through books, she will never forget them because she can link them to real experiences. Moreover, engaging in this group motivated participant A’s passion to study more about music therapy in depth. Participant A reported that “I realized I should study hard. I want to study about countertransference and projection and would like to know why people have ambivalent feelings. I became curious about them.” Participant B also shared her previous doubts. “I had not been convinced of music therapy. I had even thought about taking a leave of absence from school……. However, by participating in the group, I came to be reassured.” She further stated that her sense of identity as a music therapy student solidified as a result. Participant D stated, “As I know it [music therapy], I can talk about it more professionally to people who want to receive music therapy.” Regarding their clinical practicum, Participants A and D reported they came to understand their clients with increased empathy. Participant A began to see what occurs underneath rather than judging only the surface of clients’ reactions or behaviors. She said, “Most of my clients have intellectual disabilities. So, they tend to say whatever they want to say without filtering. I had often wanted to immediately point out when clients said something that could hurt other friends. However, I don’t want to criticize that inclination anymore.”

Two of the participants stated that the group inspired them to prepare more client-centered sessions. Participant A’s statement reflects their newly developed attitudes as music therapy students: “While participating in group music therapy, I came to think that I should prepare client-centered sessions for my clients. I really want my clients to have a meaningful time [in the session].” Participant B reported that she used to prepare music that was easy for her to play on the piano before, but now she plans more client-centered sessions and considers what would be more effective for her clients.

## Discussion

This study yielded four themes and six categories regarding personal development, professional development, and musical experiences. The results of this study had much in common with a recent qualitative study by Jackson and Gardstrom (2012), which explored undergraduate music therapy students’ experiences in group music therapy. Both studies found that students benefited from this form of group interaction and from exploring their feelings through music, reporting increased insight about themselves and more confidence in their profession of music therapy. However, there were distinct differences between these two studies, demonstrating deep cultural differences between these two populations. In the current study, conducted in Korea, students discussed how silence and a non-directive group had both been challenging, but also beneficial. Students also discussed difficult family relationships, a taboo topic in Korean culture. Moreover, because the concept of psychotherapy is deeply rooted in European and North American culture, the Korean students’ experience in group therapy was intrinsically a cross-cultural one. Therefore, in this discussion, the author will integrate the findings of each theme with the literature on this topic, addressing personal, professional, musical, and cultural contexts.

The first theme *Discovering one’s self* described Korean music therapy students’ personal development and consisted of two categories: “What it means to be me” and “Learning about self.” Participants reported that they developed a sense of self through group music therapy and increased their self-understanding. This change manifested in participants viewing themselves as individuals, and identifying feelings and thoughts. These changes are typical goals in personal therapy, especially in psychodynamic and psychoanalytic approaches, which aim to explore and understand personal behaviors and experiences such as “self-knowledge” and “self-discovery” (Leiper and Maltby, 2004, p. 52). These modalities also often seek to promote self-understanding, and a core goal of personal therapy is to gain insights about links among their thinking processes, emotions, decisions, with an emphasis on recognizing and addressing distorted logic (Sugarman, 2006). The following serve as exemplars of this: “more honest in expressing feelings” (participant B), faced introverted parts of herself (participant G), “an opportunity to concentrate on myself and enabled me to realize exactly what it means to be ‘me’ (participant A).” Similarly, Payne reported that trainee dance movement therapists that participated in a personal development group discovered “stronger sense of self.” This group became an opportunity to explore who they are and discover their “inner strength” (Payne, 2010, p. 205). She interpreted that members’ change as their ego strengthened (Freud, 1960 as cited in Payne, 2010), and they experienced individuation (Jung, 1989 as cited in Payne). As the participants in the present study shared that they came to understand themselves better than before, learned repeated patterns of taking on a specific role (such as being a caregiver), and identified new parts of themselves in contrast to group members. Von Haenisch (2011) indicated that trainee counselors came to have a better self-awareness, increased insight about their past, and develop more accepting views of themselves through mandated personal therapy. However, as Von Haenisch (2011) stated that the student therapists felt resistant and challenging to handle and face difficult issues during their personal therapy. It is noteworthy that participant G expressed her anxiety her increased awareness and new discovery in her life through group music therapy, found it difficult to develop a new perspective of her relationship with her mother, and “felt scared of therapy” because she worried everything in her life might change.

The second theme *Inside Group Music Therapy* refers to what participants experienced during the flow of group music therapy. This theme yielded four categories: “various responses to experiencing non-directive group,” “two different sides of experiencing silence,” “feeling a sense of safety and acceptance,” and “sharing difficult experience with one’s own family.” These categories represented participants’ impressions of the group leader, various reactions toward being in silence, the process of developing a sense of safety with other members, and intimate disclosures about family. In the first category *various responses to experiencing non-directive group*, participants expressed different expectations and frustrations to the group leader during the beginning phase of the group. Corey (2012) explains that providing structure in the early stage of the group is necessary because members may feel confused and anxious about how to behave in the group. However, these structures and directions should be balanced and progressively less structured to ensure that group members do not become dependent on the leader. When participants’ expectations were not met, they seemed confused about how to proceed rather than feeling capable of deciding for themselves. During this stage participants appeared frustrated and even angry at the leader. One participant was unfamiliar with group therapy, sharing anxiety that “it is not like my time was set in 10 min [to do an activity] or improvisation. How we participated changed a little bit every time. Everything was new.… When we were doing nothing, I wanted her [the leader] to make a decision for us” (Participant A). Participant B stated in the interview that “It would have been easier and more comfortable if [you] had given us directions and told us what the first, second, and third activities were.” However, Payne (2010) provides a different point of view in relation to this – that trainee dance/movement therapists project their anger on the leader because it might be easier to do so than face other peers in the group because of their need to depend on each other.

However, as time passed, the participants accepted the leader’s non-directive facilitation style and the group began to find their own flow and acknowledge the benefits of having space in the group. This non-directive group enabled them to come up with their own ideas, to express their feelings, and one participant even reported feeling surprised to see “the group was flowing like water without specific directions.”

The second category, *two different sides of experiencing silence*, captured both participants’ positive and negative reactions to silence during the group. Silence can occur in the beginning phase, especially in non-directive groups, because members are unsure of how to act. This silence can then invoke worry or a lack of clarity (Corey, 2012). Silence in group therapy may also occur during beginning stages due to “uncertainty and ambiguity,” or when group members explore emotionally sensitive topics (Brown, 2008, p. 83). In the present study, remaining in silence was sometimes “uncomfortable,” but participants hesitated to break this silence because “somebody might want to talk as well.” Later on, this silence became an opportunity for participants to open and “talk about myself more authentically,” and even resulted in insights developing over feeling pressured during silence. Consistently, Regev et al. (2016) have discussed that clients have both positive and difficult experiences during silences that occur in the beginning stage of art therapy. This uneasiness is purported to be connected to the likely undeveloped therapeutic relationship at the start of any therapeutic process. Silence evoked feelings of loneliness, nervousness, and discomfort that was difficult at the beginning of therapy when “the bond between the client and the therapist is not yet deep or strong enough.” However, clients in their research reported silence functionally served to “process things emotionally and mentally” during art therapy. Correspondingly, Gans and Counselman (2000) claimed that silence in group therapy provokes anxiety but also can present as an opportunity to “find oneself.” Silence was served as a space for the projection of specific personal experiences from the past. Indeed, participant F recalled a difficult childhood experience during the period of the silence where her mother did not speak when she was angry at the F. In sum, at the beginning phase of the course of a therapeutic group, silence can be difficult for members. While the leader can mitigate this by providing more structure, leaders can also effectively use silence to allow “clients to reflect, take responsibility, get into their feelings, and continue what they were thinking about” (Hill et al., 2003, p. 520).

In group therapy, building up trust is crucial because it enables the members to be more accepting in the group and feel safe. Group cohesiveness leads members to disclose more deeply, to reveal their personal difficulties honestly, and to view themselves through other members (Corey, 2012). Moreover, intimacy can be developed when the members share their own demands and personal problems knowing that others have similar problems helps group members to feel a sense of belonging to the group (Corey, 2012). As group music therapy in the current study evolved the participants built up trust, became cohesive, and felt a sense of safety. The two categories in the second theme—“feeling a sense of safety and acceptance” and “sharing difficult experience with one’s own family”—are related to group cohesiveness and a sense of trust. One participant expressed this metaphorically as it being like “sharing with friends” and reported “I was able to talk about and accept myself.” It also encouraged students to take risks in revealing their personal issues as one participant described that “I did not have any regret and fear at that time.” Toward the end of the course of the group, the participants began to share their family history and let themselves be vulnerable in the group. This was a very emotional time for the group that shared their grief and anger toward their families. As the group discussed their personal stories members felt empathy for each other and felt a sense of belonging. One participant described this experience as “sharing secrets with friends.” Yalom and Leszcz (2005) explains that “universality” in the group is developed when the members began to reveal and notice that they share similar pains, which helps members to feel that they belong and are included. Group cohesiveness also means that “acceptance and understanding among members may carry greater power and meaning than acceptance by a therapist” (p. 63).

The third theme *musical experience as a way to explore emotions* introduced participants’ emotional experience through projective use of music in free improvisation. Aigen (2014, p. 42) explained that “music facilitates emotional expression by bypassing internal taboos” in psychodynamic music therapy. Priestley (1987, p. 22) stated that music enables participants to access both conscious and unconscious feelings, and that improvised music plays a role as a direct connection to “emotional language.” In the present study, music as a non-verbal and abstract medium enabled participants to express and explore their feelings and thoughts and served as a cathartic experience that helped them to process their feelings. For example, participants were able to identify and articulate their feelings, expressed feelings more easily, and became aware of suppressed feelings. These outcomes demonstrate music’s capacity to reflect the “client’s emotional state” in the co-creation of music with a therapist (Turry, 1998, p. 161). Bruscia (1987, p. 561) highlights the importance of this non-verbal way of expressing emotion, with improvisation playing a role as “safe, acceptable way of expressing conflicts and feelings that are difficult to express.” For example, in the present study, expressing anger by beating the drum became a safe container to hold one participant’s rage that appeared to her as an uncontainable and totally destructive. This even “turned into a pleasant moment” and it appeared as if music provided a needed distance to protect herself as well as aided the processing of her anger.

However, the benefits of expressing feelings through music, conversely, created difficulties for some participants, who felt that music was “too abstract and symbolic.” One participant reported that she was able to hide her depressed feelings by improvising in an opposite mood, disconnecting from the emotional potential of the musical moment. Another participant struggled to express her feelings in music, even though she wanted to express herself in this way. She appeared to lack the connection with and comfort with her emotions needed to express herself musically. This result is supported by (Priestley, 1994, p. 136) caution that music is not only used to express “acceptable feelings,” but also can work as “a defense against an acceptable feeling or impulse.”

The last theme *What learned through books became real* referred to the academic and clinical development of taking part in music therapy. The firsthand experience of personal therapy as a client has an experiential learning aspect for students training to be therapists. It was a parallel process that the students in this group became more confident in the therapeutic strength of music therapy by experiencing personal changes as clients. The participants reported that they were able to witness how music works therapeutically through actual experience as a client and were motivated to study psychodynamic music therapy after observing the dynamics of transference and countertransference during their group experience. These experiences solidified their professional identity as a music therapist and enhanced students’ confidence as professionals. Participants also reported developing more non-judgmental empathy for clients. These positive aspects of experiential learning through a therapeutic experience as a client are supported by numerous studies: Payne (2010, p. 207) found that a professional development group gave trainees confidence that as dance/movement therapists they were on “the right path for them.” Bellows (2007) explained that trainees not only “enhanced their sense of professional identity” but also “most clearly valued their former therapist as a professional role model.” Bellows found that participants reported personal therapy as having the greatest influence on their own work as a therapist, as well as having a positive impact on their interpersonal relationships. Similarly, Jackson and Gardstrom (2012) indicated that undergraduate music therapy students who were engaged in a short-term group therapy were encouraged to translate their experience to their own clinical work. Trainees who experienced short-term group therapy reported an increased desire to prepare for sessions in a more client-centered way (Probst, 2015a; Lim and So, 2017). So (2017) found that student music therapists reported that undergoing personal therapy was one of the most significant ways that they learned to empathize with their clients and be more available emotionally for them. Moreover, trainee therapists came to understand “how it had felt when they were on the receiving end” (Probst, 2015a) and were able to make a quick connection to their clients through their own personal therapy (Probst, 2015b).

Overall, in the present study, Korean music therapy students experienced personal changes, clinical development, and emotional exploration in music through being engaged in group music therapy. Even though the researcher explained to participants that they might benefit in the form of increased self-knowledge, personal insights, and professional awareness before they provided consent to participate, their actual experiences in the group might differ because none of them were previously involved in group music therapy. Gaining new self-awareness, improved group interaction skills, and better access to their feelings were consistent with typical client benefits. This study is unique, however, in that it demonstrates the ways in which in group music therapy provided both personal growth and experiential learning for music therapy students of this cultural background. As music is a crucial therapeutic medium in music therapy, prospective music therapists need to trust the therapeutic strength of music. Furthermore, observing an experienced therapist’s techniques can provide a rich and vivid professional development opportunity.

### Reflection of Korean Culture in Group Music Therapy

The researcher did not directly ask the participants about their cultural perspectives while engaged in the group or any of data resources. However, it was patently necessary to examine the cultural elements present because Korean students experienced a group music therapy that was modeled on practice and theory stemmed from Europe and the United States and the researcher trained and practiced in personal therapy in the United States.

Lately, multiculturalism in psychotherapy has received attention throughout the field of psychotherapy and counseling. As “the dominant models of psychotherapy tend to be grounded in a monocultural perspective” it is essential to face cultural differences due to the possibility that these “interfere with treatment effectiveness” (Comas-Díaz, 2018, pp. 562–563). There are distinctive differences as American culture focuses mainly on people’s independence and the cultivation of an inner self (Markus and Kitayama, 1991) while Korean culture values the ideas of Confucianism which emphasizes a “proper relationship” and “harmony” among people and hierarchy (Tien and Olson, 2003). Hofstede stated that group-oriented culture emphasizes “strong, cohesive ingroups, which throughout people’s lifetime continue to protect them in exchange for unquestioning loyalty” (Hofstede, 1991, p. 51).

Considering the strong influence of Confucianism on Korean culture, it might be a natural response that the Korean music therapy students in this present study were confused and frustrated with the leader’s non-directive style during the early phase of the therapy. This frustration was reflected in the theme “various responses to experiencing non-directive group.” The author viewed participants’ reactions as stemming, in part, from a cultural difference, as the facilitator had been educated and experienced personal therapy in the United States. Some studies have shown that Korean clients preferred counselors that used a highly directive approach with suggestions that offer solutions to problems as opposed the non-directive style of counselors (Kang, 1993; Kim et al., 2002; Li and Kim, 2004). Furthermore, the expectation of the group leader to make decisions for them is connected to the view of the leader as an authority figure. This was reflected in the participant A’s statement “A leader is a leader. In Korea, [a leader] should give 99%.” Other Korean participants in this study expected the group leader to guide them with clear directions. For example, one participant wanted the leader to make decisions or directly tell them what to do in the group. When their expectations were not met, one participant thought “the group leader was not engaged with us.” Kwon (2009, p. 121) indicated that Korean immigrant women who participated in a music therapy group also reported they expected a strong leader with charisma, with participants’ expectations stemming from the fact that “Korea is a very hierarchical society.” Joo (2009) reported that Korean clients projected their expectations (of “teacher” and “superiority”) on their counselors fostering a dependency in the clients on the counselors. This expectation manifested due to the “strict social hierarchy and formalism.” However, Qian et al. (2001, p. 53) assert that this hierarchical relationship might became an obstacle for “developing [their] own resources” and considered it a form of resistance. Later, Korean music therapy students in this current study reported that non-directive style of group was beneficial to fostering their autonomy: “If there was a fixed direction from the beginning of the group, I would have tried to fit my thoughts in that direction and it would have resulted in dishonest results [or responses].” Similarly, Joo and Park (2016) explained that non-directive therapy can be helpful to aiding to clients express themselves particularly when aggressive responses or intense moments were not inhibited, and they felt trusted. Li and Kim (2004) reported that non-directive counseling sessions enable Asian American clients to independently decide “the appropriate course of action.” Kim et al. (2008) also acknowledge the dependency of Korean clients on therapists but emphasized the need for an “independent “self. That in the long-term, directive therapy may not promote real growth in Korean clients. Therefore, Korean counselors need to acknowledge the difficulties associated with satisfying the expectations of “direct guidance and a dependent relationship,” while at the same time encouraging Korean clients to develop the autonomy and strength to change (Joo, 2009).

#### Cultural Examination on Self-Disclosure

Even though clients’ self-disclosure is considered the product of successful counseling, this can be challenging for clients from Asian countries (Zane and Ku, 2014) because they worry about compromising their social status and want to hide the shameful aspects of themselves (Kim et al., 2008). Disclosures mainly involved secrets that were the most difficult to reveal, including familial history, sexual identity, sexual experiences that have never been told to others and negative feelings toward counseling or the counselor. Clients reported that they recognized the necessity of disclosing these secrets and they believed their counselors were trustworthy but simultaneously also felt “extreme fear and shame” (Han and O’Brien, 2014). Inhibitions toward self-disclosure were reflected in the categories sharing difficult experience with one’s own family and *musical experience as a way to explore emotions*. Some participants did not want to talk about their unhappy family or parents’ divorce because of fear of judgment: “I was not being honest [about my family] and wanted to present only a happy side.” Another participant shared that it was the first time that she had talked about her parents’ divorce, and that she still felt hurt by her family situation. This finding is supported by Lai and Tsai (2014) as cultures in East Asian countries emphasize “parental authority, filial piety, and saving face” and prescribe that children should “not wash dirty linen in public,” to avoid causing shame for their family and the loss of social standing (Lai and Tsai, 2014). This cultural stigma stigmatizes attending counseling as it may “bring disgrace to family members” due to the group-oriented nature of the culture (Lai and Tsai, 2014, p. 497). In addition, asserting their thoughts and expressing their feelings can be negatively perceived in Korean culture. Chung (2013) explained that Korean women were raised to repress emotions such as hatred, anger, and resentment as well as their joy, desire, and will because they belong to men. Some of the participants in this current study appeared to be uncomfortable and felt guilty when expressing themselves. The first theme *Discovering one’s self* also included participants’ cultural view of personal changes, with one participant labeling herself as “selfish” when she began to express her feelings and thoughts. Participant D also stated that “speaking up my voice” might interrupt other members and “would try not to stand out myself.” When For participant G, before expressing her anger by beating the drum, she requested to the group to play as a whole to cover her sound. She was satisfied when angry music turned into pleasant moment. This reaction can be viewed both a reflection of the expression of personal needs that also presented itself through cultural expectation of restraining self-expression.

Therefore, clinical suggestions have been formulated concerning the therapists’ attitude and way of interacting with the Korean clients have been discussed in a number of studies. Han and O’Brien (2014, p. 545) explained that therapists working with Korean clients need to exercise patience and seek to develop a strong therapeutic alliance prior to expecting “the real work of therapy to commence.” Joo (2009) stated several key elements are necessary to assist and enable clients to experience the therapeutic relationship as less hierarchal, including adopting an “inviting” attitude, developing “collaborative and egalitarian relationships,” and fostering “trust and honesty.” Moreover, therapists need to maintain consistency between their expression of emotion and action, avoid behaving authoritatively, and expressing their thoughts simultaneously (Joo and Park, 2016). Kim and Park (2016) indicated that therapists’ humor, honesty, self-disclosure, and empathy can be helpful. In sum, maintaining a non-authoritarian manner and horizontal relationship could be a key to ensuring the benefit of Korean clients in any therapeutic experience.

## Limitations and Recommendations for Future Studies

This study is firstly limited by linguistic issues as all the data was collected in Korean and were translated into English by the researcher. This might have possibly resulted in the participants statements being conveyed differently from what they had originally meant. In this qualitative case study, 8 weeks of group music therapy was provided to Korean music therapy students due to their academic load. However, Klein (1985) argued that 12–15 consecutive sessions of short-term group therapy can be effective, and it is appropriate that each of these sessions last between 45 and 90 min. Therefore, in the future, research interested in further exploring this topic should increase the numbers of sessions of the group.

It may arguably also be important in future research to include more male participants in the group to explore how their dynamics would manifest in the group. When two male students dropped out from the group due to the family circumstances, both participants did not directly notify the researcher of their decision. During the process of the data analysis and discussion with advisor and committee, it was wondered whether the male students might be angry at the researcher as a leader for not being as strong as they expected, or they might have felt uncomfortable expressing themselves in a female majority group. Therefore, it would be significant to examine the cultural expectations of Korean male participants and perhaps adapt the group to deal with potential cultural barriers.

As discussed in the literature review section, only few studies have previously been conducted on the role of experiential therapy on music therapy trainees in Korea. The results of the present study could therefore promote the importance of including personal therapy and experiential learning in Korean music therapy training programs, especially for programs oriented toward a psychodynamic approach. Like Gardstrom and Jackson’s (2011) survey of American Music Therapy Association program coordinators, in which participants shared ethical concerns regarding requiring personal therapy, the present study invites questions regarding the ethicality of requiring personal therapy in Korean music therapy training programs.

## Conclusion

Consistent with the evidence from the previous studies reported, the findings of this study reveal tangible personal, clinical, and musical developments in the participants. The group music therapy served as an opportunity for Korean music therapy students to discover their ownership of self, to gain support from the group, to share their wounded parts, to connect with music, and develop clinical competency. For music therapy educators, this qualitative case study provides further insight and knowledge into how the experience of personal therapy helps music therapy students’ growth because therapeutic work requires personal maturity and a deepened understanding of their clients. Finally, the current study is significant as it provides information on how culturally laden elements of the therapeutic process can be reexamined and applied appropriately for Korean clients.

## Data Availability

All datasets generated for this study are included in the manuscript and the supplementary files.

## Author Contributions

The author confirms being the sole contributor of this work and has approved it for publication.

## Conflict of Interest Statement

The author declares that the research was conducted in the absence of any commercial or financial relationships that could be construed as a potential conflict of interest.
